# Breaking the Hydrogen Bond Barrier Reversibly: Toward Ultradrawable Polyamides

**DOI:** 10.1021/acsapm.5c00426

**Published:** 2025-05-26

**Authors:** Milo Gardeniers, Nils Leone, Roy Kneepkens, Amy van Diepen, Jörn Droste, Michael Ryan Hansen, Sanjay Rastogi, Jules A.W. Harings

**Affiliations:** † Aachen-Maastricht Institute for Biobased Materials, 127355 Maastricht University, P.O. Box 616, Maastricht 6200 MD, The Netherlands; ‡ 5211 Fontys University of Applied Sciences, P.O box 347, Eindhoven 5600 AH, The Netherlands; § Institute of Physical Chemistry, 9185 University of Münster, Corrensstr. 30, Münster 48149, Germany; ∥ King Abdullah University of Science and Technology, 4700 KAUST, Thuwal 23955-6900, Saudi Arabia

**Keywords:** polyamide 6, ions, crystal structure, ultradrawable, mechanics

## Abstract

In polyamides, hydrogen bonding and conformations of amide motifs are strongly influenced by pH, ions and their concentration, and water molecules and their structure. To fulfill the physical requirements for ultradrawing of polyamide 6, we first complete our fundamental insight into the role of water, ions, and polyamide 6 crystal structures on the concept of reversible shielding of hydrogen bonds. The reversible shielding depends on a complementary superchaotropic effect of anions and the kosmotropic effect of cations, locally affecting the structuring and interactions of water. We show that in the presence of large halogen anions, specifically polyiodides, crystallization from the random coil state or during crystallographic reorganization is suppressed by hydrophobic hydration. Among the cations, hydrated lithium and calcium cations promote the formation of polyiodides, specifically I_3_^–^. The small size of lithium cations entails high diffusivity with water molecules, retrospectively effectively shielding the hydrogen bonding in the crystals. Upon reorganization of the conformationally distorted β phase upon heating and close to the boiling point of water, ions promote gel formation. The gel can be extruded and shaped, e.g., into monofilaments at 85 °C, and at room temperature, it can be stretched to a draw ratio of 25 to secure chain orientation. After immersion in water to remove the ions and restore the amide–amide hydrogen bonds, postdrawing and drying render high anisotropy, oriented chain crystals of high perfection, and tensile modulus and strength up to ∼19 × 10^3^ and ∼1140 MPa, respectively. The process holds potential in achieving extended chain crystals desired for ultimate mechanical properties.

## Graphical abstract

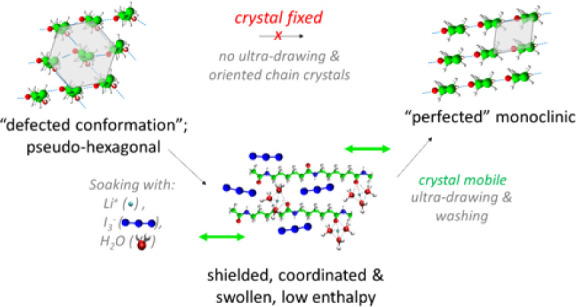

## 1 Introduction

Ever since the rise of polymers and fibers made thereof, high-performance fibers have been used in demanding mechanical and lightweight applications.^1^ High modulus and high strength combined with low density make these materials outperform other anisotropic construction materials on a weight basis. The common feature of high-performance polymer fibers is the high degree of molecular and structural orientation along the fiber axis—anisotropy. Secondary interactions are generally too weak to account for high moduli and strength, but due to the molecular and structural overlap at sufficiently large length scales, the cooperative effect of the secondary interactions prevents intermolecular and intercrystalline slip transferring mechanical stress to the covalent bonds in the main chain of the polymers under load. The high energy required to increase bond lengths and angles is the origin of the high modulus and high strength. Treloar described the idea of single-chain deformation theoretically.^2^ His calculations disclose that the deformation of an individual fully extended polyamide and polyethylene molecule in a vacuum, which in reality does not exist, requires the deformation of bond lengths and bond angles, raising the elastic modulus by about a hundred times.^2^

The idea of polymers adopting extended chain conformations arose with the introduction of artificial, man-made polymer fibers.^3^ Already in 1932, Carothers and Hill^3^ pondered the following question: “what molecular requirements are prerequisite for the manufacturing of a high-performance fiber balancing processability and performance?” They pictured a perfectly oriented fiber to consist of a single crystal in which the long molecules are ordered in an array parallel to the fiber axis. Regrettably, in the absence of external fields such as flow or mechanical stress, polymers with conformationally flexible backbones do not retain a uniaxially oriented structure due to entropically driven relaxation.^4,5^

Nevertheless, “freezing” of polymers with high anisotropy, i.e., evading entropy-driven chain relaxation, has been realized by the introduction of conformationally rigid aromatic macromolecules with the right configuration to facilitate cooperatively strong intermolecular secondary interactions.^6^ Examples are poly­(*p*-phenylene terephthalamide) (PPTA), poly­(pyridibisimidazole) (PIPD), and poly­(*p*-phenylene benzobisoxazole) (PBO), renowned for their roles in high demanding applications.^1,5,7,8^ Due to the intrinsic conformational chain rigidity, the small difference in entropy between the crystalline and liquid states raises the melting temperature beyond the degradation temperature. Due to this so-called intractability, the macromolecules have to be mobilized using solvents. For solubilization, the strong intermolecular forces need to be overcome and often demand harsh organic solvents such as concentrated sulfuric acid in the case of PPTA. Even in solution, the contour of the molecule is hardly affected as the rigidity dominates, dictating a lyotropic liquid crystalline state that readily promotes molecular and structural orientation in extensional flow. Although the molecular weights are limited,^9^ the cooperative secondary interactions between molecules and structural planes facilitate adequate stress transfer from the macroscopic scale to the polymer chains, resulting in a modulus that approaches the calculated elastic modulus in the case of PPTA.^6^

For conventional synthetic polymers, such as nylons, polyesters, and polyolefins, the situation is very different. Here, the fiber modulus is nearly 1 order of magnitude smaller than the crystal modulus.^10^ One example of a flexible polymer where entropy-driven chain relaxation can be overcome is ultrahigh molecular weight polyethylene (UHMWPE). In this scenario, the realized elastic modulus is close to the theoretical modulus.^11^ The high modulus relies on effective intermolecular stress transfer between polyethylene chains in a fully extended chain conformation. The structure is obtained after postdrawing of solution-spun fibers below their melting temperature, but above the α-relaxation temperature where intracrystalline chain dynamics exist.^12,13^ Polymers with intracrystalline chain dynamics are classified as “crystal-mobile”. Once in an extended chain conformation, effective stress transfer via intermolecular van der Waals forces, in the order of 5 kJ/mol or less, demands ultrahigh molecular weights.^2^ To process polyethylene solutions of such high molar masses, the number of entanglements per chain is reduced using dilute solutions.^14^ Another approach is via synthesis, evading the need and recovery of excess amounts of solvent. Here, disentangled chains are realized upon using the right catalyst, pressure, and temperature.^15–18^ Polymerization below the dissolution temperature of the polymer enforces the growing polymer chain to crystallize before entangling. The reduction of entanglements either by solvents or synthesis combined with postdrawing below the melting temperature are today industrially adopted technologies that yield extended chain structures and moduli approaching the theoretical crystal modulus of PE, being 220 × 10^3^ MPa.^11^

In the case of polyamide 6, the theoretical modulus was calculated to be 311 × 10^3^ MPa.^11,19^ The intermolecular hydrogen bonding between adjacent amide motifs is postulated to introduce high thermal resistance and overcome creep while being melt-processed without harsh organic solvents. However, ultradrawing of aliphatic polyamides into extended chain crystals is hindered by the cooperative energy of the hydrogen bonding between adjacent crystalline stems, the so-called hydrogen bond barrier.^20^ From the moment of anisotropic deformation, the reduced entropy due to the orientation of the polyamide chains induces crystallization. The hydrogen bonding defines the crystal to be “crystal-fixed”, preventing the cooperative translation of chain segments through the crystal lattice to ultimately form extended chain crystals.^20,21^ The maximum draw ratio is therefore determined by the presence of hydrogen bonds acting as physical constraints and is limited to 5, independent of temperature.^20^ The pseudohexagonal crystal phase, existing in quenched samples and above the Brill transition temperature, is often marked as a conformationally mobile phase. Although one may hence anticipate ultradrawability of the pseudohexagonal phase, it locks into the monoclinic α phase upon uniaxial deformation.^22,23^ In the past, scientists have attempted to temporarily shield the amide–amide hydrogen bonding enabling intracrystalline chain dynamics and molecular orientation into extended chain crystals during ultradrawing using ammonia,^24^ iodine,^25,26^ inorganic salts,^27^ Lewis acid–base complexes like GaCl_3_,^27–29^ and polar aprotic solvents, all with limited success. Najafi et al. provided a comprehensive review of melt and solution spinning processes, disclosing methods to utilize nanofillers, plasticizers, and copolymer strategies to interspace PA chains, weaken H-bonding, and increase chain mobility for enhanced draw ratios.^4^ Scalability of the reversibly plasticized systems was challenged by limited solubility and incomplete removal of the additives used to preserve the oriented state.

Inspired by natural silk spinning, where pH and ion exchange processes mediate fibroin solubility and the transitions into liquid crystalline and ultimately β-sheet crystalline structures in the so-called aquamelt,^30–34^ we aim to introduce additional degrees of freedom and develop a processing route for the ultradrawing of polyamides using the high aqueous solubility and facile exchange of lithium- and calcium-based halogenic salts. With increasing molarity, lithium and calcium ions promote the formation of polyiodides, which are chaotropic in nature and entropically driven to intercalate in hydrophobic regions of the supramolecular structure of cellulose, facilitating the exfoliation of cellulose nanofibers from their micrometer ensemble (Gardeniers et al, submitted). Similarly, Tashiro et al. reported recently the crystal structures of intercalated PA 6,6 model compounds and polyamide 6 and 6,6 polyiodide complexes using two-dimensional wide angle X-ray diffraction, polarized Raman spectroscopy, and DFT calculations.^35^ The structural analysis reveals, as recently disclosed in cellulose,^36,37^ the swelling of the unit cell, conformational changes in the CH_2_–amide linkages, and shielding of the direct amide–amide hydrogen bonding by water molecules and I_3_^–^ and/or I_5_^–^. As the study focused on the traditionally used KI/I_2_ solutions to form polyiodides, the distinct effect of the hydration ability of the cations, as disclosed in our earlier work, remains unexplored. To ultimately define and test the optimum conditions for ultradrawing of polyamide 6, we first complete our physical understanding on the role of water, ions, and the initial polyamide 6 crystal structure on the reversible shielding of polyamide 6 hydrogen bonding. We study polyiodide intercalation varying the hydration ability of the cations via crystallization or crystal transformation of polyamide 6 in water. The use of superheated water as a solvent for polyamides has been demonstrated, where the dissolution temperature (*T*_d_) ranges between 150 and 240 °C, depending on conformational disorder, the crystal phase, and amide density.^38–41^ Crystal phases marked by conformational disorder like the pseudohexagonal β phase tend to dissolve and reorganize in water at temperatures just below 100 °C, minimizing the loss of molar mass and mechanical properties. During crystallization and crystal transformation in the presence of water, water molecules were reported to reside in and plasticize the crystal lattice of polyamide 6. From a conformational, structural, and thermo-mechanical perspective, we aim to understand the effect of iodide intercalation while varying the hydration ability of the cations in polyamide 6 and test reversible shielding of the hydrogen bond barrier and facilitate ultradrawing, promoting the formation of extended chain crystals. The spinning process is foreseen and underlying the conceptual methodological approach in this research as presented in Scheme 1.

**Scheme 1:**
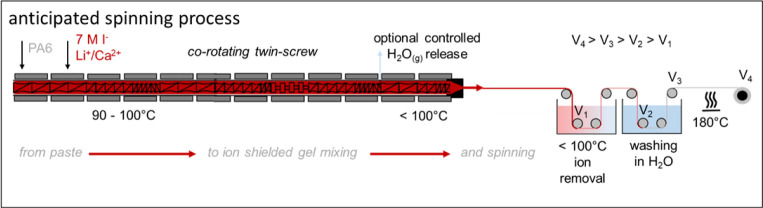
Schematic Representation of the Anticipated Spinning Process of Polyamide 6 (PA6) That Is First Mixed and Soaked with 7 M LiI Solution and Successively Heated and Compounded to Form a Homogeneous ion Shielded PA6 Gel, Optionally Vented to Remove Excess of Water, Spun and Postdrawn While Ions and Water Are Removed and Hydrogen Bonds Are Restored

## 2 Experimental Section

### 2.1 Materials and Sample Preparation

Commercial polyamide 6 (Akulon F136) was supplied by DSM. Salts if hydrated in the hydrated form (LiCl, LiI, CaI_2_, NaI, and KI) and iodide were purchased from Sigma-Aldrich. The weight-average molecular weight of PA 6 was 74,000 g/mol as measured by gel permeation chromatography (GPC) against PMMA standards. To determine potential changes in molecular weight upon various (hydro)­thermal treatments, GPC was carried out on a PSS SECurity GPC system using Agilent 1260 Infinity instrument technology. The apparatus was equipped with a PFG combination precolumn and two PFG combination microcolumns. Distilled 1,1,1,3,3,3-hexafluoroisopropanol (HFIP) containing 0.019% sodium trifluoroacetate was used as an eluent using a 0.3 mL/min flow rate at 40 °C. The sample weight was kept constant at 5 ± 0.2 mg. The degradation dependence on temperature and time was studied by exposing PA6 in closed capillaries, with or without ions, to 90, 120, 150, and 180 °C for 1, 3, 10, and 30 min, respectively, in an in-house-designed pressure cell.

For ease in processing and wetting, the granulate as received was cryogenically milled using Fritsch Pulverisette 14 equipped with a 0.5 mm sieve. The sieved fraction with a particle size ranging from 0.4 to 0.5 mm was used for the premix, GPC, and DSC experiments. A premix of 20 wt % PA6 cryo-grinded powder and 80 wt % 7 M LiI was added to a 20 mL Biotage microwave reactor vial, and the premix was vigorously stirred. The vials were closed with a septum and transferred to a reaction vessel at 125 °C for 30 min. This process will be referred to as the gel route. The obtained gel was fed to an Xplore twin-screw microextruder of 5 mL in volume at a screw speed of 50 rpm at a temperature of 85 °C equipped with a fiber die of 0.75 mm. Note that the extrusion temperature of the polyamide gel is well below the conventional extrusion temperature of PA6, which is 250–260 °C. Mixing was performed for 3 min. The transparent yellow extrudate with a diameter of 0.75 mm was drawn using a Zwick Roel retroline Z100 tensile tester equipped with a 1 kN load cell. No preload was applied. The draw ratio was studied using elongation rates between 100 mm/min and 1000 mm/min, respectively. To deshield the amide moieties and restore the amide–amide hydrogen bonding, the drawn extrudates were sprayed with water at different draw ratios. All the experiments were performed at room temperature. As a reference, an extrudate directly from the extruder was collected and washed in water. The elongated filaments were immersed in excess water, approximately 60 L, and held under tension by the addition of a 20 g weight for 24 h at 20 °C. Next, half of the filaments were post-drawn over a Köfler bench (Wagner and Munz, type WME) at about 180 °C. To remove the excess of water and water potentially trapped in the crystal lattice,^25^ the filaments under tension were dried at 80 °C for 1 h and 160 °C for 24 h *in vacuo,* respectively. All successive steps are schematically illustrated in Scheme 2.

**Scheme 2:**
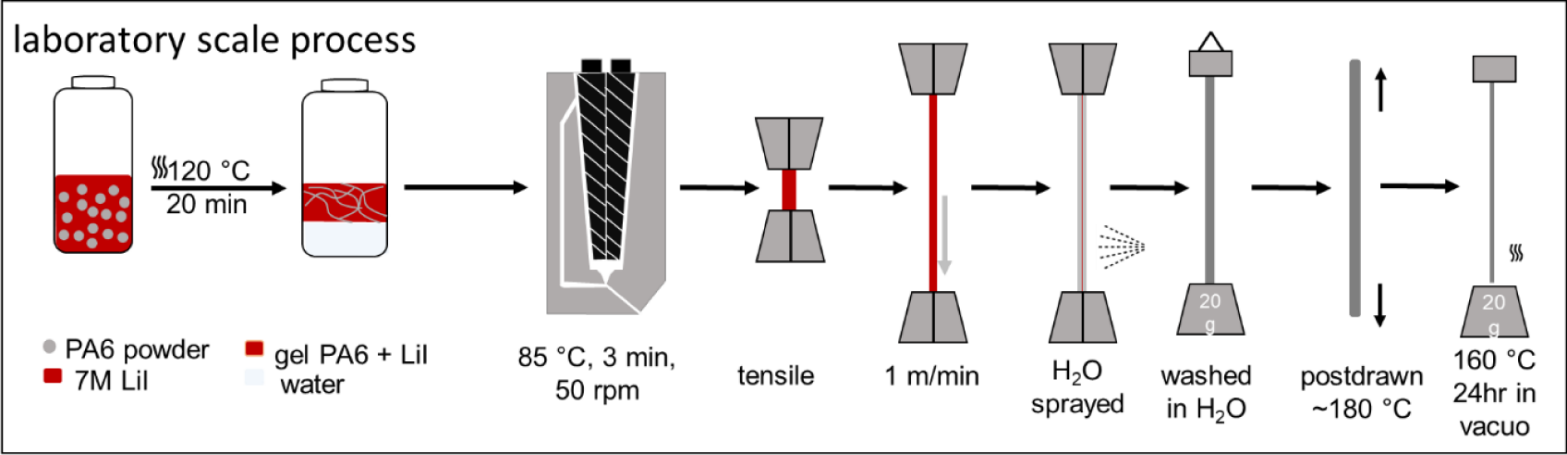
Schematic Representation of All Successive Steps of the Laboratory Scale Process, Including All Elements of the Anticipated Large-Scale Spinning Process

### 2.2 Differential Scanning Calorimetry

Differential scanning calorimetry (DSC) was performed on a TA Instruments Q2000 DSC apparatus operating under a nitrogen atmosphere. The dissolution of polyamide 6 was studied in superheated water, with or without the presence of ions.^40,42,43^ High-volume DSC pans that sustain the desired pressures were used. Dissolution temperatures of PA6 samples with different controlled crystallographic states were recorded by immersing 20 wt % of polyamide 6 in aqueous solutions and exposed to a temperature program ranging between 30 and 200 °C at a rate of 10 °C/min in a nitrogen atmosphere. An isothermal period of 3 min was applied to ensure an equilibrium state at the temperature limits. Dissolution and crystallization temperatures were determined by the peak maxima and minima of the DSC thermographs, respectively.

### 2.3 Thermogravimetric Analysis

The residual weight of shielded LiI gel was studied by using thermogravimetric analysis (TGA) using a TA Instruments Q500 apparatus. Approximately 20 g of the sample was heated at 10 °C/min to 700 °C under a nitrogen atmosphere.

### 2.4 Fourier Transform Infrared Spectroscopy

Fourier transform infrared (FTIR) spectra were recorded on a PerkinElmer Spotlight 400 spectrophotometer equipped with an attenuated total reflectance setup (μATR) using a diamond crystal. The samples were recorded from 4000 to 750 cm^–1^ with a spectral resolution of 2 cm^–1^ and an average of 128 scans in the reflectance mode.

### 2.5 Solid State Nuclear Magnetic Resonance (NMR)

The ^13^C­{^1^H} CP/MAS experiments were done at 11.74 T on a Bruker Avance NEO (11.74 T, ν_L_(^1^H) = 500.38 MHz, ν_L_(^13^C) = 125.83 MHz) using a 4 mm H/F/X MAS DVT probe equipped with magic-angle gradient coils. 50 μL of ionic solutions of LiI, LiCl, KI, CaI_2_, and NaI with increasing molarity were pipetted into 4 mm HR-MAS rotors, equipped with an upper spacer made of Teflon, and sealed with a Kel-F screw and cap. Adamantane was used as an external reference for calibration of radiofrequency fields (ν_rf_(^1^H) = 50.0 kHz and τ_π/2_ = 5.0 μs) and referencing the chemical shift scale (δ (^1^H) = 1.85 ppm and δ (^13^C) = 29.47 ppm).

### 2.6 Dynamic Mechanical Thermal Analyses

The thermomechanical performance of PA6 filaments obtained from the gel route was measured on Mettler Toledo DMA 1 in a tension setup using a temperature range from −100 to 150 °C, a heating rate of 3 °C/min, and a frequency of 1 Hz. A preload of 0.01 N and an amplitude of 10 μm were used.

### 2.7 Draw Ratio and Tensile Testing

The influence of the draw ratio on the mechanical properties of undried and postdrawn dried PA6 filaments was investigated using a Zwick Roel retroline Z100 tensile tester equipped with pneumatic clamps and a 1 kN load cell. Samples were tested in 5-fold, employing a deformation rate of 50 mm/min.

### 2.8 Wide-Angle X-ray Diffraction (Cu Kα)

2D wide-angle X-ray diffraction (WAXD) was carried out by using a SAXSLAB Ganesha diffractometer with Cu Kα radiation (λ = 0.145 nm). The beam center and 2θ-range were calibrated via the diffraction pattern of silver behenate. The conversion of 2D into 1D data was performed using Saxsgui v2.13.01 software. The CPI^44^ is thus calculated using eq 1, where Ω = 0.194.

1 $$\mathrm{CPI}=\frac{\left(\frac{d_{200}}{d_{002}}\right)-1}{\Omega }$$

### 2.9 Scanning Electronic Microscopy

Surfaces and cross sections of PA6 filaments, dried and postdrawn dried, were visualized by scanning electronic microscopy (SEM) using a Jeol JSM-IT2000 microscope. Samples were submerged in liquid nitrogen and cut with a razor blade. Prior to analysis, samples were coated with a layer of gold before viewing.

## 3 Result and Discussion

### 3.1 Influence of Ions on Dissolution and Crystallization in the Superheated State of Water

The dissolution (temperature) of polyamides in superheated water depends on the hydrogen bonding efficiency and mobility of water molecules and amide moieties in the polyamide crystals in a specific temperature and pressure window.^19,43,45,46^ Upon heating polyamides, the rotational momentum of thermally induced aliphatic gauche conformers mobilizes the amide moieties, weakening the amide–amide hydrogen bonding considerably although their attractive forces prevail partially.^39,42^ In a closed system, the increased polyamide mobility in the crystal allows relatively mobile water molecules of the superheated state to enter the polyamide crystal. These water molecules perturb the amide–amide hydrogen bonding, leading to crystal refinement and solubilization dependent on the initial crystallographic state, temperature, and chemical environment, e.g., dictated by ions.^38,43^

Depending on the hydration ability, valence, and atomic radius of the cations, hydration shells with varying metal ion–water binding energies and coordination numbers are formed. Figure 1a discloses *T*_d_ of PA6 in the superheated state of water, varying the molarity of the solutions irrespective of its complex structure. Strong suppression of *T*_d_ of PA6 in water is only observed for CaI_2_ and LiI. In fact, Deshmukh et al. explained the reduced *T*_d_ of PA46 in the triclinic form in the superheated state by means of temperature-dependent solid-state ^1^H HR-MAS NMR spectroscopy.^40,41^ The effect of temperature on the chemical shift of the water protons was more pronounced with increased molarity. It was concluded that the decreased intermolecular hydrogen bonding efficiency and increased mobility of water are promoted by the ions, lowering *T*_d_. However, a clear understanding of the mechanism discriminating the role of the hydrating cations and nonhydrating anions remained ambiguous.

**Figure 1:**
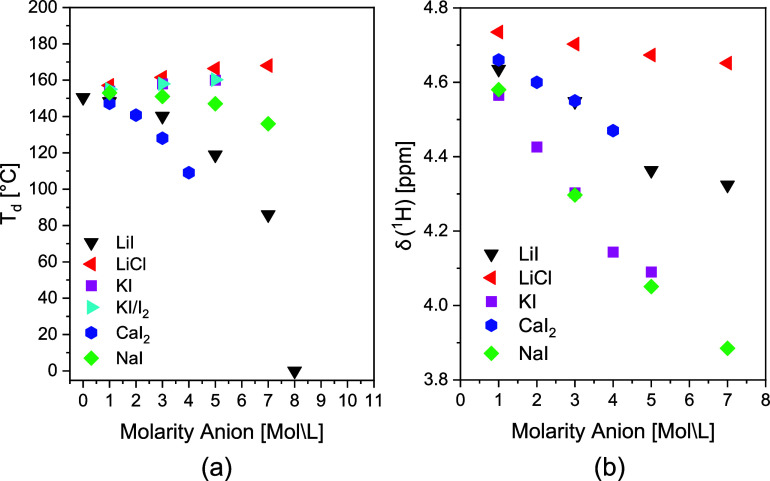
(a) The dissolution temperature (*T*_d_) of PA6 as a function of anion molarity and (b) the ^1^H chemical shift in HR-MAS NMR spectroscopy signifying the intermolecular hydrogen bonding and mobility of water molecules for systematically varied combinations (non)­hydrating ions.

CaI_2_, LiI, and KI/I_2_ promote polyiodide formation in water, whereas KI and NaI do not.^37^ Polyiodides like I_3_^–^ and I_5_^–^ are stronger chaotropes than I^–^. Are polyiodides solely responsible for the decrease in *T*_d_ of PA6? And, how important is the hydration ability of freely diffusing cations shielding the intrasheet hydrogen bonding as observed in our earlier work on polyamide 46 ^41^ and recently observed in the disruption of the cellulose supramolecular structure?^37^ As *T*_d_ of PA6 is hardly affected in KI and KI/I_2_ solutions, the cations must have a significant effect on lowering *T*_d_ of PA6. Whereas Ca^2+^ and Li^+^ form tightly bound hydration shells, K^+^ and Na^+^ do not.

In Figure 1b, changes in the intermolecular hydrogen bonding efficiency and mobility of water molecules were traced by a decreased chemical shift of the water protons as a function of molarity using solid-state ^1^H HR-MAS NMR spectroscopy. Iodide is the largest chaotropic anion of the halogens, resulting in the lowest ion–H_2_O bond energy, −10.3 ± 0.3 kcal/mol, compared to −14.7 ± 0.6 kcal/mol for Cl^–^–H_2_O bond energies.^47^ Large chaotropic anions are nonpolar in nature. Water molecules form a surrounding cage-like solvation shell to maintain maximum H_2_O–H_2_O hydrogen bonding that in fact is stronger than I^–^–H_2_O.^47,48^ As the radius of chaotropic ions increases beyond a certain critical value, the efficiency of hydrogen bonding in the first coordination shell becomes disrupted due to geometrical restrictions. Specifically, the first solvation shell of iodide exhibits only 2.5 hydrogen bonds between water molecules on average (against 3.4 in pure water), lowering the ^1^H chemical shift.^45,48^ Comparison of the ^1^H chemical shifts in aqueous LiCl and LiI solutions confirms the stronger chaotropic nature of iodide. According to the Hofmeister series, an increase of the cationic radius, here from Na^+^ to K^+^, will increase the chaotropic behavior as observed by a further decrease of the ^1^H chemical shift of water protons. With the increasing cationic radius from NaI to KI, *T*_d_ of PA6 returns to 150 °C—identical to *T*_d_ without ions.^45,49,50^ *T*_d_ even increases beyond 150 °C with increasing KI and KI/I_2_ molarity as the population of bulk water molecules decreases, while in the case of KI/I_2_, the polyiodides are promoted. It is evident that, like in cellulose, the large hydrodynamic volume of hydrated (kosmotropic) cations swells and shields the amide–amide interactions of the intercalated PA6-polyiodide complex as crystallographically described by Tashiro et al.^35^ They tentatively concluded that upon immersion in concentrated KI/I_2_ solutions, I_3_^–^ and I_5_^–^ reside within the crystal lattice of PA6 and coordinate to the amide protons.

*T*_d_ of PA6 is strongly affected by the chosen combination of ions, as witnessed in DSC. Figure 2 shows the influence of LiI with increasing molarity on the dissolution process as followed during the first and second heating. The relationship between molarity and *T*_d_ of the monoclinic phase with increasing molarity, Figure 2a, has been explained for PA 46 by solid-state NMR spectroscopy before.^40^ With increasing molarity, Li^+^ and I^–^ ions mobilize the water molecules, reaching the same mobility representative of the superheated state at decreased temperatures. Water molecules and ions migrate into the crystalline domains, forming a clathrate in which the amide–amide hydrogen bonding is shielded, reducing *T*_d_ and ultimate enthalpy of dissolution as disclosed in Figure 2c,d. The formation of tri-iodides and their higher shielding ability in clathrates that form above 3 M^37^ explain the change in slope. Upon cooling from the homogenized dissolved state, clathrates are formed with reduced *T*_d_ and enthalpies in the second heating. In fact, at 7 M LiI, PA6 remains solubilized.

**Figure 2:**
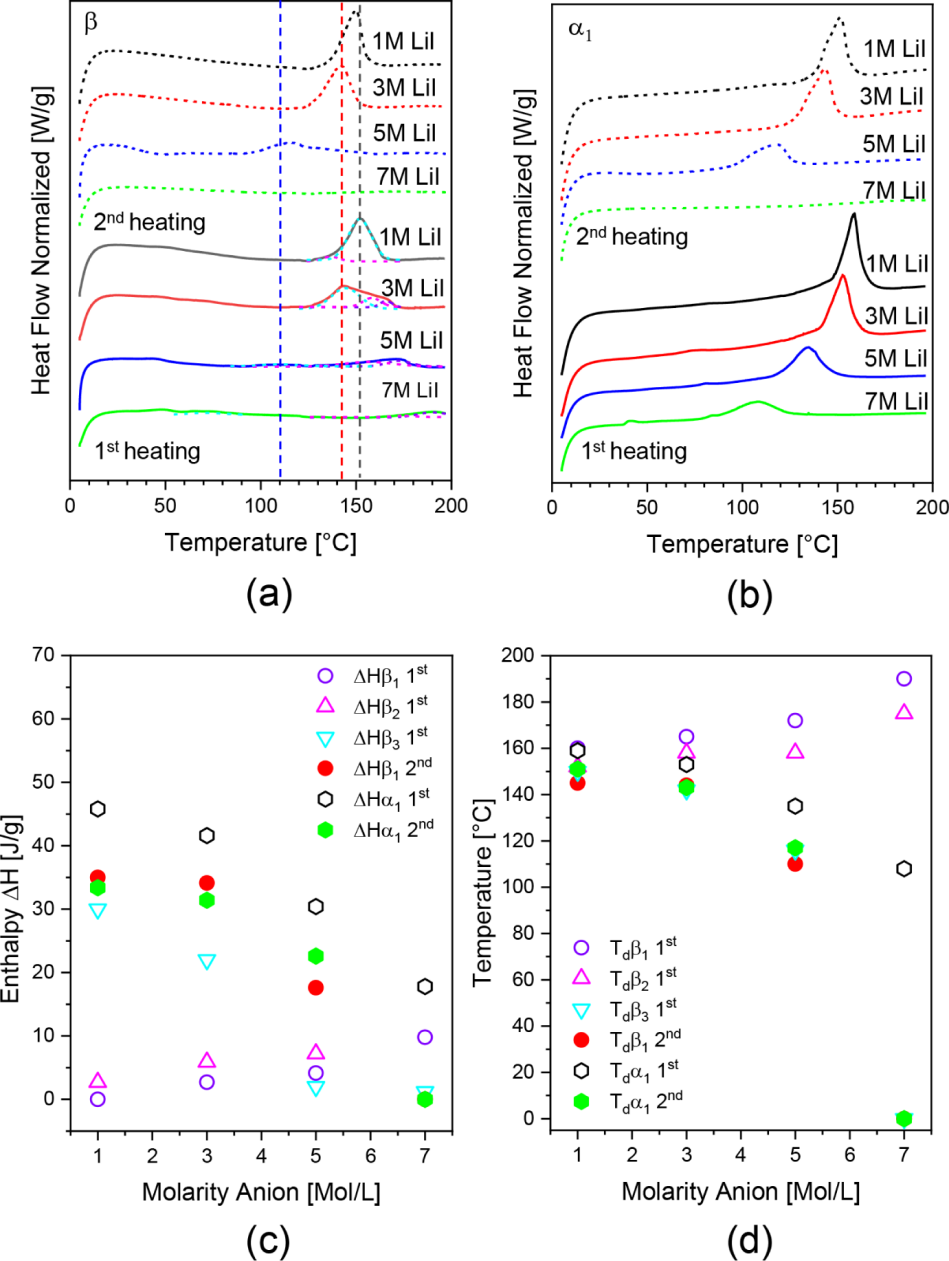
DSC thermograms of PA6 treated in water and aqueous LiI ranging in molarity. Visualizing melting-point suppression of PA6 with increasing LiI molarity (a). The influence of LiI on the dissolution temperature (*T*_d_) of different PA6 crystal phases, monoclinic, melt quenched (pseudohexagonal) (closed symbols), and corresponding enthalpies (open symbols) (b). Suppression of the dissolution (closed symbol) and crystallization temperature (open symbol) of PA6 with increasing LiI molarities (c). Lines are guides for the eye.

The effect of molarity on the dissolution of PA6 in the pseudohexagonal β form is remarkably different, Figure 2b. The dissolution peaks in the first heating constituted multiple endothermic events. Deconvolution shows a minimum of three distinct peaks. One peak, *T*_d_β_3_ first in cyan, shifts to lower temperatures, matching *T*_d_ of the clathrates *T*_d_α_1_ second and *T*_d_β_1_ second in the second heating (Figure 2c). The other two peaks, being *T*_d_β_1_ first and *T*_d_β_2_ first, shift to higher temperatures, although the enthalpy of these fractions is low. In fact, the enthalpy of dissolution of these peaks decreases with increasing molarity. By means of in situ solid-state NMR spectroscopy and combined SAXS/WAXD studies on PA6 and water, we reported that during the structural β → α reorganization in the presence of water, water molecules appear within the crystal lattice.^38^ Similarly, the presented DSC studies (Figure 2) reveal that during the β → α reorganization, also the ions, specifically tri-iodides, enter the crystals, forming clathrates with weakened amide–amide interactions. The two peaks with an upward shift in *T*_d_ also reveal that not all amide moieties are shielded. The unshielded, but crystallographically and energetically refined PA6 crystals are marked by a high *T*_d._ ^38^ The immersion of PA6 in LiI solutions of high molarity may entail extrusion and postdrawing of PA6 into extended chain crystals at temperatures below the β → α transition.

Prior to the attempt, the influence of 7 M LiI on hydrolytic degradation under the employed superheated state conditions must be known. Figure 3 and Table S1 depict the weight-average (Mw), number-average molecular weight (Mn), and polydispersity index (PDI) of PA6 exposed to different ionic solutions in the superheated state of water for different temperatures and time-scales. Exposure of PA6 to heat and 7 M LiI for a period longer than 3 min reduces the molecular weight. The molecular weight of PA6 remains unaltered in the temperature range of 90–150 °C for 1–30 min. Considering the changes in Mw and PDI in Table S1, it is evident that from 150 °C onward, Mn decreases and PDI increases. Hence, the water- and ion-assisted reduction in processing temperature below 150 °C suppresses undesired hydrolytic effects and preservation of Mw and Mn that is of importance to the ultimate mechanical properties.

**Figure 3:**
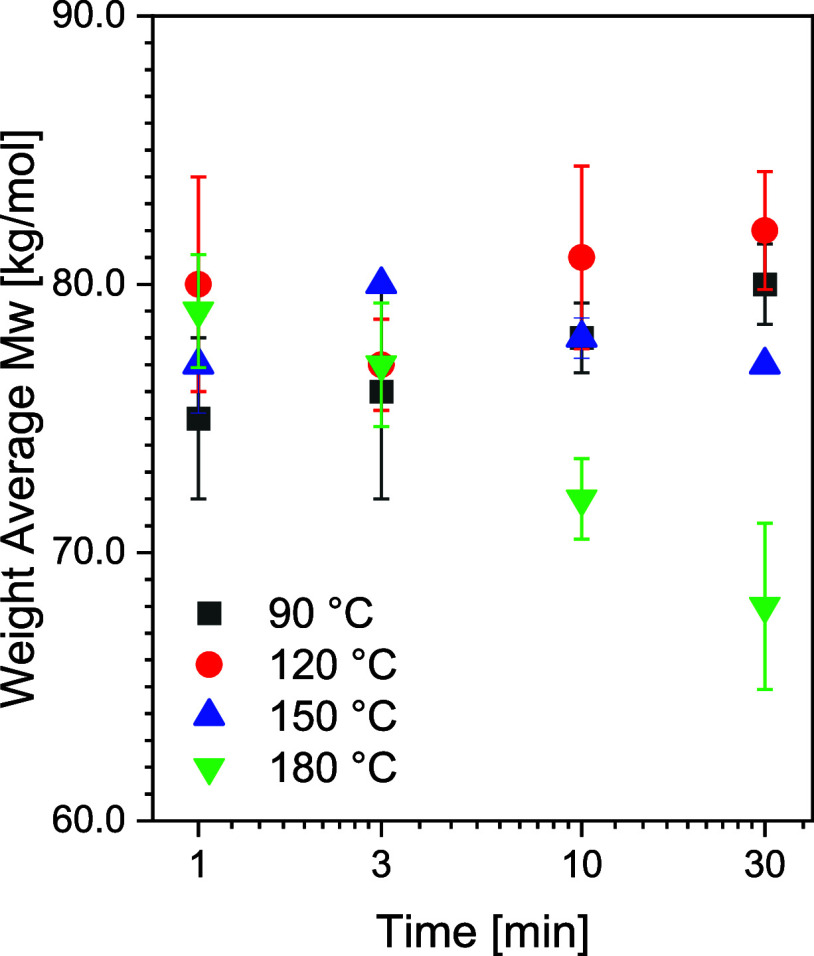
Overview of the weight-average molecular weight obtained via GPC of 7 M LiI treated at different temperatures and time intervals.

### 3.2 Shielding of Amide Hydrogen Bonding and Aqueous Extrusion and Postdrawing

The technical impact of the explored route to ultradrawing depends on the reversibility of the ion-induced shielding of the amide motifs. To understand the state of shielding and deshielding as a function of LiI molarity, PA6 extrudates (see Section 2) before and after washing were analyzed by using FTIR spectroscopy. Note that washing to remove the ions while preserving orientation occurred under a continuous load. The FTIR spectra of the extrudates with increasing LiI molarity are compared in Figure 4 to spectra of PA6 as prepared in the α, β (Figure S1), γ crystal phases^51^ and in the melt state. Assignment of the most relevant vibrational modes of the various states is listed in Table 1. Crystallization of PA6 during processing in the presence of water promotes the formation of the α phase,^38^ represented by the red spectrum in Figure 4. Upon increasing the LiI molarity to 3 M, blue shifts were observed for the NH stretch and amide II overtone bands to 3298 cm^–1^ and 3096 cm^–1^, respectively. Simultaneously, two shoulders appear for the amide I and amide II at 1652 cm^–1^ and 1548 cm^–1^ with red and blue shifts, respectively. This trend is opposite to the weakening of amide–amide hydrogen bonding as observed at elevated temperatures in the crystalline and/or melt state.^52^ In fact, Magill and Matsubara noted identical shifts as those for 3 M LiI in KI/I_2_ induced PA triiodide complexes,^53^ accompanied by new peaks in the 1200–900 cm^–1^ region (here 990 cm^–1^). With respect to the α phase, the CH_2_ scissoring and C–C skeletal vibrations in the regions of 1478 to 1416 cm^–1^ and 1125 to 1029 cm^–1^ remain unaltered. At 5 M LiI, the amide I and amide II bands show different asymmetries, and a red shift is observed for amide I to 1620 cm^–1^, whereas the amide II band shifts to 1548 cm^–1^. Next to that, red and blue-shifted amide I and amide II shoulders with increased absorbance at 3 M LiI show opposite shifts on the expense of the dominating absorbance in the α-phase. The wavenumbers at 5 M LiI, being 1652 and 1542 cm^–1^, respectively, match the melt state of PA.^22^ The red shift for amide I indicates an increase in bond length due to the shielding of the amide–amide direct interactions at ionic strengths from 5 M LiI onward.^54,55^ At lower wavenumbers, the CH_2_ scissoring bands at 1478 and 1416 cm^–1^ for the α-phase decrease in intensity and disappear at 5 M LiI, while 1456 and 1440 cm^–1^ augment in agreement with the KI/I_2_ complexes.^53^ In terms of molecular conformations, this means that from 5 M onward, the amides are rotating out-of-plane yet retained in the crystalline state. Besides, the all-trans methylene conformers transition to a distorted (gauche) state as observed in the melt. It coincides with the loss of the monoclinic phase and the corresponding vibrational modes at 952 and 930 cm^–1^. Above 5 M LiI, the broadening of the N–H stretch band around 3291 cm^–1^ paralleled by an increasingly apparent O–H stretch bend signifies weakening of the hydrogen bonds and an increasing amount of water in the system.

**Figure 4:**
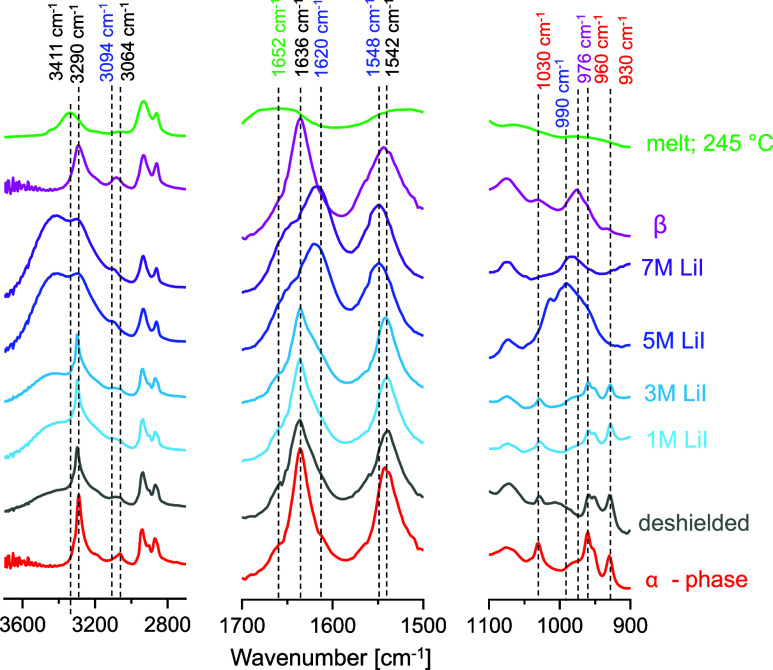
FTIR spectra of PA6 LiI extrudates with increasing molarity in the range of 1–7 mol/L (blue/purple), in the deshielded state (gray), and compared to the crystallographic monoclinic α (red) and pseudohexagonal β structure (pink), and in the melt state (in green).

**Table 1:** Most Relevant Vibrational Bands of Different Crystallographic Polyamide Morphologies and the Influence of 3 M LiI and 5 M LiI on Their Shifts

vibrational band							
α	3 M LiI	5 M LiI	deshielded	β	γ^51^	melt	vibrational mode
3290	3298	3294	3298	3292	3285	3344	**NH stretch**
3092	3094	3103	3091	3082	3087		**Amide II overtone; CN stretch and CNH bending** in-plane; constrained
3062	3060		3060			3060	**Amide II overtone; CN stretch and CNH bending in-plane**
2946							**CH_2_ antisymmetric CH stretch;** α_N_CH_2_ constrained
2940	2936	2934	2936	2932	2929	2932	**CH_2_ antisymmetric CH stretch**
2870	2868		2868				**CH_2_ symmetric CH stretch;** α_N_CH_2_ constrained
2854	2852	2862	2854	2860	2847	2862	**CH_2_ symmetric CH stretch**
		1652				1652	**Amide I:C**=**O** **stretch**
1636	1636		1636	1638	1641		Amide I; H-bonded
		1620^53^			1623		**Amide I; ion shielded**
		1548			1549		**Amide II: CN stretch and CNH bending in-plane; ion shielded**
1542	1542		1540	1544			Amide II; constrained
		1514			1513	1514	Amide II
1478	1478		1478				α_N_CH_2_ scissoring; constrained trans conformation
				1476			α_N_CH_2_ scissoring; nonconstrained trans conformation
1464	1464	1464	1464	1462	1467		CH_2_ scissoring non amide vicinal; conformationally disordered, amorphous
		1456			1460	1456	**CH_2_ scissoring; mobile gauche conformation**
1434				1434			CH_2_ scissoring; conformationally disordered, amorphous
		1440			1440	1440	**–CH**_**2**_ **–CONH–CH**_**2**_ **–** **rotation out of trans conformation** ^56^
				1420			α_CO_CH_2_ scissoring; nonconstrained trans conformation
1416	1418		1416				α_CO_CH_2_ scissoring; constrained trans conformation
1372	1374	1360	1372	1370	1366	1358	**Amide III coupled with hydrocarbon skeleton; CN stretch and in-plane NH deformation**
1292	1292	1284	1292			1274	Amide III: CN stretch and CNH bending in-plane coupled to skeletal carbon (α_CO_CH_2_)
1266	1266		1264	1262	1264		Amide III; crystalline
1202	1202		1200	1204			Amide III
1125	1119		1123	1118	1121		C–C skeletal stretch
1110		1114	1110		1116	1112	C–C skeletal stretch; amorphous
1030	1029		1029	1032			C–C skeletal stretch;
		990					Amide IV, amorphous^53^
				976	975		Amide IV: C–CO stretch
960	960		960		962		Amide IV: C–CO stretch
930	928		930				Amide IV: C–CO stretch
730	730		730	728	725		Amide V
692	688		692				Amide V

Washing in excess of water, forcing the ions to diffuse out of the polyamide phase leads to restoration of amide–amide hydrogen bonding, named as the deshielded state in Figure 4 and Table 1. The CH_2_ scissoring vibrations of the deshielded state are identical to the methylene trans conformation of the monoclinic α structure. While exposure to KI/I_2_ solutions induces the α → γ transformation, the LiI complexation (shielding) is fully reversible although the match of the NH stretch, amide II overtone(s), and CH_2_ stretch vibrations to the 3 M LiI sample suggest some residual shielding effects. This shielding is not per se directed to residual ions as crystallization and structural reorganization in water may also lead to entrapped water molecules in the crystal lattice.^38,42^

The drawability of 7 M LiI-shielded PA6 extrudates is shown in Figure 5. Following Newton’s second law of motion, i.e., force is equal to mass multiplied by acceleration,^57^ increasing the deformation rate increases the force to flow and the ultimate draw ratio as observed in Figure 5a. Once the maximum force is exceeded, fixation of stretched and oriented polymer molecules in the drawing direction is hypothetically induced by ion removal. While being strained using a deformation rate of 1000 mm/min, water spray was applied at draw ratios ranging from 2 to 10. Figure 5b reveals that at the moment of spraying, the force and thus resistance against extensional deformation rapidly increase by the restoration of amide–amide hydrogen bonding. Successive DMTA experiments reveal the thermo-mechanical behavior of the LiI-shielded, sprayed, washed, dried, and melt-processed (ref) extrudates (Figure 5c,d). The peak maxima of the loss modulus (*E*″) indicate that with respect to the PA6 reference filament, *T*_g_ in the 7 M LiI-shielded state decreases from about 75 to – 48 °C (Figure 5d). Below *T*_g_, the “frozen” aqueous phase entails a high storage modulus (*E*’), ∼5.500 MPa, of the 7 M LiI sample as extruded and sprayed (Figure 5c). In comparison to the 7 M LiI-shielded sample, the sprayed sample shows a second *T*_g_ at about 0 °C and a two decade increase of *E*’ above both glass transition temperatures. This indicates that upon spraying, restoration of amide–amide hydrogen bonding induces crystallization of PA6 only in a fraction of the sample, generating a core–shell morphology in which the core contains a high concentration of ions and the shell contains a low concentration. The core–shell morphology gives structural integrity to the fibers. After extensive washing and successive drying at 160 °C under vacuum and continuous load, both *T*_g_ and *E*’ below and above *T*_g_ restore to values of the melt-processed reference extrudate (Figure 5c). These results conclusively demonstrate that shielding of amide moieties by 7 M LiI suppresses the crystallization of PA6, enables draw ratios up to 4–5 times more than conventionally achieved in melt processing, and can be reversed completely upon excessive washing.

**Figure 5:**
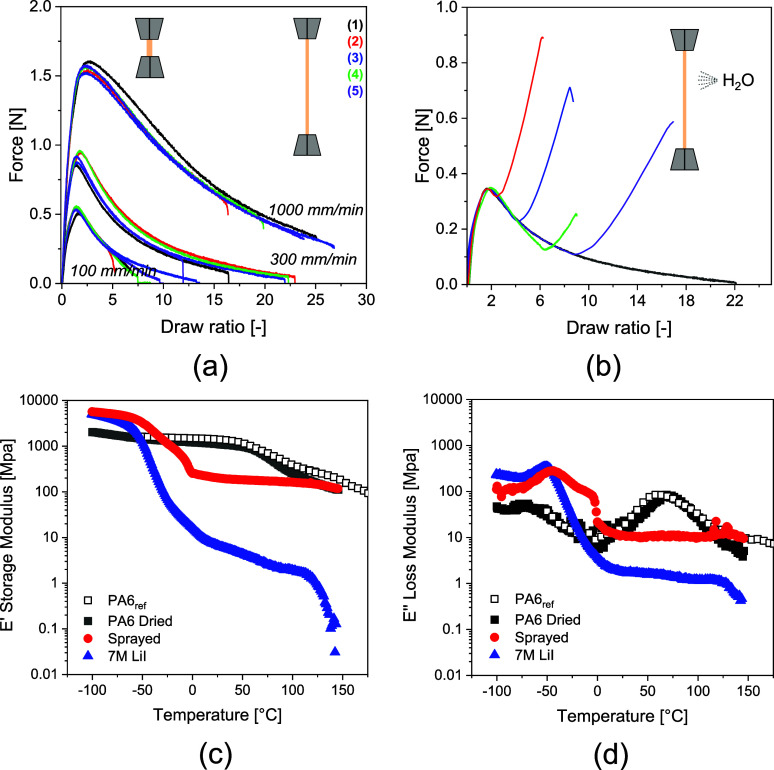
Force as a function of draw ratio demonstrating the influence of (a) deformation rate and (b) timed water spraying on shielded PA6 extrudate during drawing. (c) Storage *E*′ and (d) loss *E*″ modulus of the PA6 extrudates without any treatment (PA6_ref_), and in gel-phase with 7 M LiI, sprayed with water, and subsequently washed and dried as a function of temperature.

### 3.3 Structure Development on Drawing

Inefficient removal of shielding agents played a negative role in the reversible nature of previously reported plasticization strategies.^25,26^ To achieve fibers with high modulus and high strength, timing of the complete washing during postprocessing is crucial. High draw ratios, achieved at 1000 mm/min (Figure 5a), are of specific interest in this work. 7 M LiI shielded PA6 extrudates were drawn repeatedly to draw ratios of 25 times, monitoring consistent force diagrams to prevent the introduction of morphological or structural defects. Such defects lead to deviations in the force diagram and/or early breakage. In an attempt to minimize molecular relaxation of the noncrystalline domains by entropic forces, the drawn extrudates (25 times) were first subjected to water spray before terminating the drawing. The sprayed samples were washed under constant load in excess water for 24 h. Effective removal of residual water from both the amorphous and crystalline states was ensured by successive drying at elevated temperatures close to the Brill transition in vacuo^38^ and under load. The tensile behavior of the sprayed, as-drawn, and postdrawn PA6 filaments is compared in Figure 6h. Specific values describing the tensile behavior are summarized in Table 2. The stress–strain response obtained for the PA6 extrudate with a draw ratio of 25 shows not the expected high modulus and high stiffness of a highly drawn filament despite the high crystal perfection index of the α phase as recorded by WAXD and introduced by Murthy et al.,^44^ Figure 6a,b. Detailed analysis of the WAXD and SAXS detector images of the sprayed and washed 7 M LiI PA6 filament reveals medium anisotropy (Figure 6b,d,g), the presence of a long period (Figure 6f), and overall diffuse scattering typically caused by residual ions. Specifically, the anions rich in electrons act as scattering centers. It is likely that those small traces of ions facilitate the postdrawing and the unique structure observed by SAXS and WAXD, Figure 6c–e. Although the CPI is slightly lower (0.9 instead of 1.0), Figure 6a, the anisotropy and lateral crystal dimensions clearly increase with the loss of ion-induced scattering and the long period, Figure 6f. The WAXD of the drawn, sprayed, and postprocessed filaments shows signals in the meridional plane that originate from the structural repeat of the lattice planes along the *c*-axis. However, no signal in the meridional plane of the corresponding SAXS pattern is observed, which is a direct effect of the lack of a long period by fading structural boundaries, i.e., a sufficiently large amorphous phase that separates the oriented crystals along the filament axis. The absence of the long period demonstrates the effective reversible breaking of the hydrogen bond barrier via the Li^+^ polyiodide complexation and fixation of the polyamide chains in extended chain crystals. As a result, the tensile modulus and strength increase from 2,035 and 70.3 MPa to 18,700 MPa and 1,143 MPa, respectively (Table 2). Optimum postdrawing conditions in real spinning are likely to refine the structure throughout the fiber volume, increasing the stiffness and strength further.

**Figure 6:**
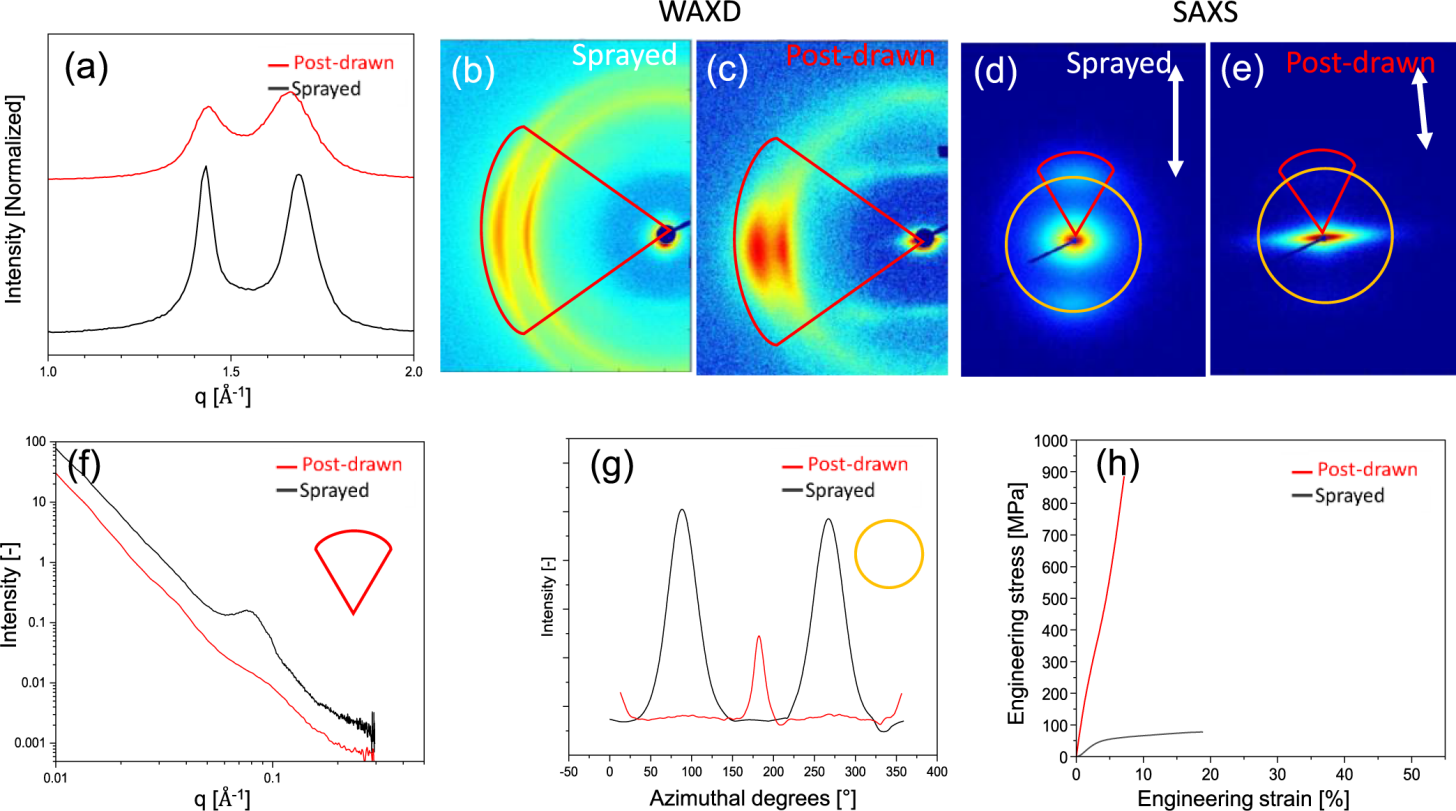
Structure analysis of sprayed and postdrawn PA6 filaments with an initial draw ratio of 25×. (a) Azimuthal integration of the equatorial diffraction signals in the WAXD patterns of (b) sprayed and (c) sprayed and postdrawn PA6 filaments. SAXS patterns of (d) sprayed and (e) sprayed and postdrawn PA6 filaments and the (f) azimuthal integration of *q* in the meridional planes and (g) the radial integration at *I*(*q*)_max_. The tensile behavior of the PA6 filaments sprayed and postdrawn or not is given by (h) the engineering stress–strain diagram.

**Table 2:** Modulus, Strength at Break, and Elongation at Break of PA6 7 M LiI Filaments with 25× Draw Ratio at Different Stages Postprocessing

sample	modulus (MPa)	strength at break (MPa)	elongation at break (%)
gel state (as extruded)	0.66 ± 0.0	-	2451.0 ± 131.0
sprayed, washed, and dried	2,035 ± 0.2	70.3 ± 5.7	19.7 ± 8.7
sprayed, washed, dried, postdrawn	18,700 ± 2.3	1143.2 ± 226.7	7.9 ± 1.7

Scanning electron micrographs of 7 M LiI PA6 filaments at different stages of the process, Figure 7, reveal that the removal of ions by washing and successive drying yields a porous structure. Variations in water content during processing may affect the association of ions in forming salts that upon washing dissociate, promoting the formation of pores. The same porous morphology was observed for polyamide plasticized with GaCl_3_ or CaCl_2_.^58–60^ The porosity can be overcome by reducing the diffusion path, i.e., decreasing the cross-sectional diameter of the filaments and timing of washing during postdrawing. Figure 7c,d shows that the porosity-induced surface roughness fades upon refined postprocessing. Variations in size and spatial distribution, i.e., inhomogeneity, have been reported more often after solution spinning.^4^ Pores may, but not per se, affect the physical and mechanical properties of the filaments, which have to be addressed in a future up-scaling endeavor.

**Figure 7:**
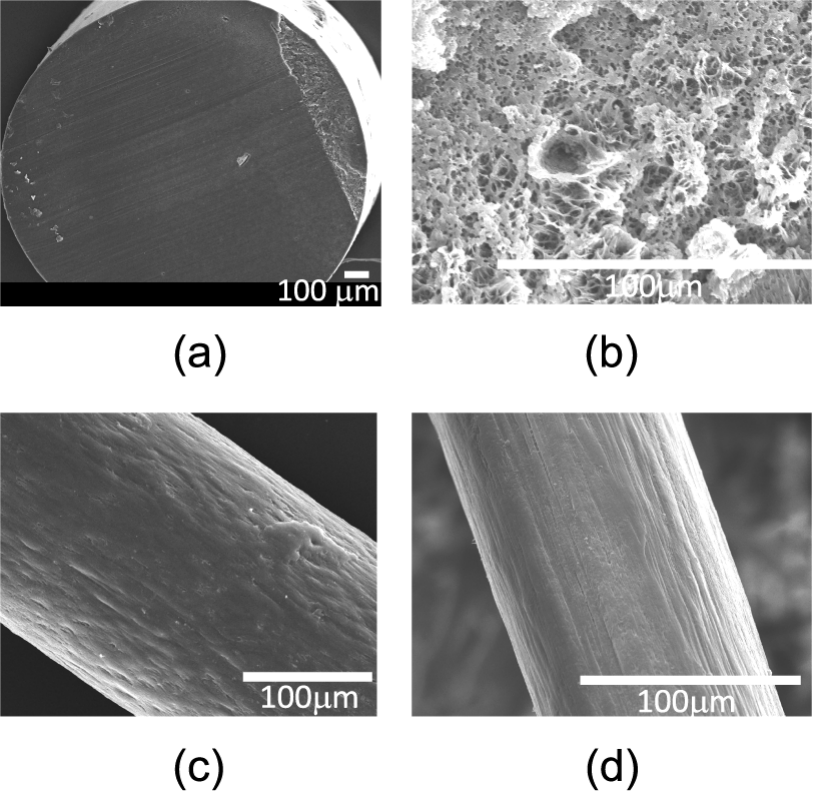
SEM images of 7 M LiI filaments (a) undrawn, washed, and dried at 80× and (b) 500× magnification and (c) 25× drawn, sprayed, and dried fibers without and (d) with postdrawing.

## 4 Conclusions

Secondary interactions play a crucial role in the uniaxial orientation of polymer chains. Depending on the interactions and the characteristic ratio of the polymers, the processing is strongly influenced. For example, while aramids require a lyotropic phase in solution spinning, the high polymer viscosity of ultrahigh molecular weight polyethylene demands a reduction in the entanglement state. Here, we conclusively demonstrated that flexible linear polyamides can be drawn uniaxially, at room temperature, by shielding the persistent hydrogen bonding motifs. With deshielding of the shielded hydrogen motifs, the mechanical properties of the uniaxially drawn fibers are recovered. Our findings show a strong influence on the cationic size in the effective shielding of the hydrogen bonding of polyamide 6. Cationic size, valence, and hydration ability on (poly)­iodide intercalation and plasticization of the polyamide 6 crystal lattice conclusively show that Ca^2+^ and Li^+^ promote the formation of I_3_^–^ and I_5_^–^ anions, respectively. Hydrothermal DSC and solid-state ^1^H NMR spectroscopy reveal that the entropic penalty of water molecules organized in hydrophobic hydration shells surrounding the anions drives I_3_^–^ and I_5_^–^ to “hide” in the apolar methylene-rich motifs of the PA6 crystal lattice. The iodides are stabilized energetically by N–H···I_3_^–^ charge transfer, i.e., hydrogen bonding. Due to the small size and cationic charge, Ca^2+^ and Li^+^ strongly bind water molecules in hydration shells, increasing their hydrodynamic volume, expanding the PA6 unit cell, and shielding the amide–amide interactions. Governed by polymorphism, the ions suppress the dissolution temperature of PA6 in water. Without ions, the energetically less stable pseudohexagonal β phase reorganizes into the more stable monoclinic α phase, but with increasing CaI_2_ and LiI molarity, the pseudohexagonal phase tends to dissolve at significantly lower temperatures. The presence of ions reduces the dissolution temperature, suppressing the hydrolysis of PA6, which is advantageous in preserving the molar mass required for processing. FTIR spectroscopy of PA6 in shielded and deshielded states after washing demonstrates complete reversibility of the hydrogen-bonded amide moieties with the removal of Li^+^ and polyiodides. Unlike traditional KI/I_2_ treatment where polyiodides are coresponsible for the irreversible induction of the γ phase, the presented reversible shielding route yields the α phase. The reduced dissolution temperature of specifically the pseudohexagonal phase of PA6 in the presence of LiI facilitates extrusion below 100 °C. The resulting extrudate is amorphous, having a glass transition temperature of −48 °C, and can be drawn up to 25 times at room temperature. Upon water spraying, with the selective removal of ions from the surface, a core–shell morphology is obtained, where restoration of the hydrogen bonding in the shell provides structural integrity to the extrudate. Under continuous load, washing in excess water, successive drying, and postdrawing close to the Brill transition temperature yield oriented chain crystals with high crystal perfection that translates into high tensile modulus and tensile strength.

## Supplementary Material



## Notes

An overview of the weight-average, number-average molecular weight, and polydispersity of PA6 samples treated with 7 M LiI at different time and temperature intervals (Table S1); FTIR spectra and WAXD patterns of different crystal structures of the PA6 polymorphs (Figure S1) (PDF)
