# Supplementary material for: Breaking the Hydrogen Bond Barrier Reversibly: Toward Ultradrawable Polyamides
Source: ACS Appl Polym Mater. 2025 May 26;7(11):6825–36. doi: 10.1021/acsapm.5c00426 (PMC12172016; doi:10.1021/acsapm.5c00426)
Supplement: Supplementary file 1 [file ap5c00426_si_001.pdf]

SUPPORTING INFORMATION

Breaking the Hydrogen Bond Barrier reversibly; towards Ultra-Drawable Polyamides

Milo Gardeniers[a], Nils Leone[a], Roy Kneepkens[a], Amy van Diepen[b], Jörn Droste[c], Michael Ryan Hansen[c], Sanjay Rastogi[a,d] and Jules A.W. Harings[a]*

[a]Aachen-Maastricht Institute for Biobased Materials, Maastricht University, P.O. Box 616, 6200 MD, Maastricht, The Netherlands. [b]Fontys University of Applied Sciences P.O box 347, 5600 AH, Eindhoven, The Netherlands. [c]University of Münster, Institute of Physical Chemistry, Correnssstr. 30, 48149, Münster, Germany. [d]King Abdullah University of Science and Technology, 4700 KAUST, Thuwal, 23955-6900, Saudi Arabia.

* Email: jules.harings@maastrichtuniversity.nl

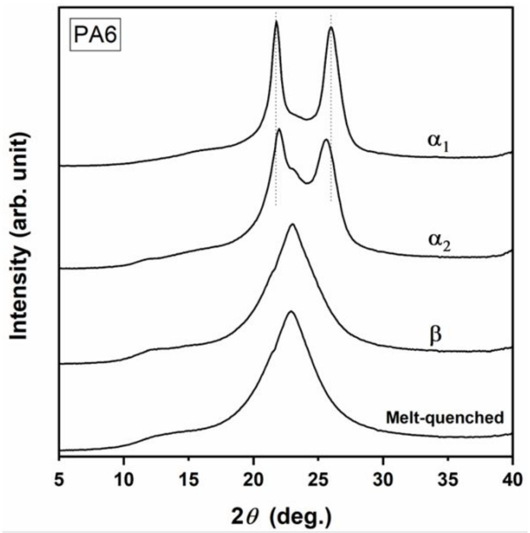
PA6
α1
α2
β
Melt-quenched
Intensity (arb. unit)
5
10
15
20
25
30
35
40
2θ (deg.)

(a)

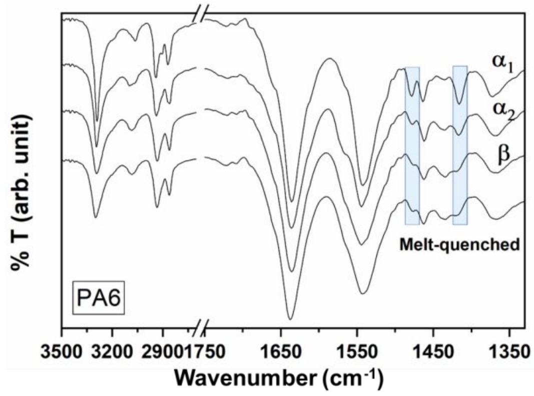
α1
α2
β
Melt-quenched
PA6
% T (arb. unit)
3500
3200
29001750
1650
1550
1450
1350
Wavenumber (cm-1)

(b)

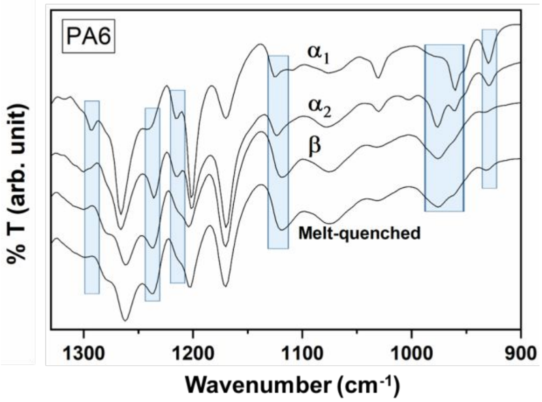
PA6
α1
α2
β
Melt-quenched
% T (arb. unit)
1300
1200
1100
1000
900
Wavenumber (cm-1)

(c)

Figure S1: **(a)** WAXD and **(b,c)** FTIR spectra and of Polyamide 6 crystal phases; namely perfected monoclinic α, defected monoclinic α and pseudohexagonal β phase.

Thermal Treatment	Mw [kg/mol]	Mn [kg/mol]	Dispersity [-]
**90 ˚C, 7M LiI**			
1 min	75 ± 3.0	30 ± 1.5	2.3 ± 0.1
3 min	76 ± 4.0	31 ± 2.1	2.3 ± 0.2
10 min	78 ± 1.3	29 ± 0.8	2.6 ± 0.1
30 min	80 ± 1.5	28 ± 1.2	2.8 ± 0.2
**120 ˚C, 7M LiI**			
1 min	80 ± 4.0	30 ± 1.2	2.5 ± 0.4
3 min	77 ± 1.7	28 ± 1.4	2.7 ± 0.1
10 min	81 ± 3.4	27 ± 2.3	2.6 ± 0.3
30 min	82 ± 2.2	29 ± 1.7	2.8 ± 0.2
**150 ˚C, 7M LiI**			
1 min	77 ± 1.8	23 ± 1.1	3.3 ± 0.5
3 min	80 ± 0.2	22 ± 0.8	3.6 ± 0.2
10 min	78 ± 0.8	23 ± 1.1	3.4 ± 0.3
30 min	77 ± 0.20	20 ± 1.1	3.9 ± 0.2
**180 ˚C, 7M LiI**			
1 min	79 ± 2.1	15 ± 1.8	5.2 ± 0.4
3 min	77 ± 2.3	19 ± 2.1	4.1 ± 0.5
10 min	72 ± 1.5	16 ± 1.7	4.5 ± 0.4
30 min	68 ± 3.1	14 ± 2.3	4.9 ± 0.3

Table S1. Overview of weight average, number average molecular weight, and polydispersity of treated PA6 samples at different time and temperatures with 7M LiI.
